# Three-Dimensional CT Planning for Anatomic Shoulder Arthroplasty: Reliability of Version Correction With Short-Term Clinical Outcomes

**DOI:** 10.7759/cureus.104284

**Published:** 2026-02-26

**Authors:** Akshar P Thakkar, Ashorne K Mahenthiran, Geneva Ringsby, Sanket C Shah, Eric M Tarkowski, Margaret Ranum, Sanjeev Bhatia, Aaron A Bare

**Affiliations:** 1 Department of Orthopaedics, Northwestern Medicine, Warrenville, USA; 2 Department of Orthopaedic Surgery, Northwestern University Feinberg School of Medicine, Chicago, USA; 3 Department of Medical Education, Lake Erie College of Osteopathic Medicine, Erie, USA; 4 Department of Radiology, Northwestern Medicine Central DuPage Hospital, Winfield, USA

**Keywords:** 3d ct planning, anatomic total shoulder arthroplasty, glenoid version, posterior augmented glenoid, shoulder arthroplasty short-term outcomes

## Abstract

Introduction: Anatomic shoulder arthroplasty for end-stage glenohumeral arthritis offers excellent outcomes for many patients. Computed tomography (CT) has become recognized as a valuable preoperative planning tool for assessing acquired bone deficiencies, such as excessive posterior glenoid version. It remains uncertain whether three-dimensional (3D) CT technology helps surgeons reliably restore native glenoid version and if version correction offers superior short-term clinical outcomes.

Methods: Fifty consecutive patients undergoing anatomic shoulder arthroplasty were enrolled in this study. A preoperative CT scan with 3D planning software was used to restore a normal range of glenoid version (0 to 10 degrees retroversion). All patients received a pegged cemented glenoid polyethylene component and underwent a postoperative CT scan to evaluate component alignment and glenoid version. Outcomes were measured by the ASES (American Shoulder and Elbow Surgeons) shoulder score and PROMIS (Patient-Reported Outcomes Measurement Information System) Global Health survey at baseline, 14 weeks, one year, and two years.

Results: Using the conventional radiological reading method, 76% of patients had their glenoid version corrected to 0-10°. Nearly all postoperative measures significantly improved at two-year follow-up, including pain, pain medication consumption, and range of motion. There were no statistically significant differences in patient outcomes when comparing glenoid version within versus outside the normal glenoid version correction range.

Conclusion: Restoring near-anatomic glenoid version is believed to be an important step to maximize prosthetic longevity. Three-dimensional CT planning software offers ways to recognize and correct abnormal versions. In the study cohort, approximately three in four patients were corrected to normal levels. There was no statistical difference in short-term surgical outcomes within or outside the desired version range.

Level of evidence: Level IV.

## Introduction

Anatomic total shoulder arthroplasty (aTSA) has emerged as the gold standard for surgical treatment of end-stage glenohumeral arthritis with an intact rotator cuff and no significant glenoid bone deformity or subluxation that fails traditional non-surgical management [1,2]. More than 40% of patients with degenerative shoulder osteoarthritis may have abnormal glenoid morphology at the time of an aTSA [3]. While many studies have demonstrated the beneficial effect of aTSA on both shoulder function and quality of life, long-term clinical outcomes rely on multiple factors, including restoring glenoid version [4,5]. Uncorrected glenoid retroversion during aTSA may lead to an increased likelihood of glenoid prosthetic loosening and early implant failure due to the rocking horse phenomenon of the glenoid component, making excessively retroverted glenoid cavities a challenging issue for surgeons.

Maximizing prosthetic longevity relies on balancing soft tissues, restoring bone anatomy, and obtaining rigid prosthetic fixation [6]. Due to generally excellent early clinical outcomes obtained with aTSA, the procedure now allows for the inclusion of broader surgical criteria and is growing in utilization [7]. With glenoid component malfunction reported as the most common cause of aTSA failure, intraoperative correction of glenoid version to within normal limits has been a focus of new innovations for preoperative planning. This new focus has been met with a variety of newly released preoperative planning software that allows for three-dimensional (3D) visualization of a patient’s native glenoid and humerus using CT scans, offering a more thorough understanding of an often complex wear pattern.

While an abnormal version can be a biplanar deformity, posterior wear is usually the primary and constant deformity that warrants correction. Version correction techniques include asymmetric (high side) anterior glenoid reaming, posterior glenoid bone grafting, or the utilization of polyethylene wedge implants. Wedged polyethylene glenoid implants (stepped or angled) offer bone preservation and the ability to correct larger defects not possible with asymmetric reaming [8]. Posterior glenoid augments may also help maintain soft tissue tension by lateralizing the glenoid. Restoring glenoid version and prosthetic seating to greater than 90% is believed to appropriately balance the load-bearing head on the glenoid component and help prevent early aTSA failure due to loosening of the polyethylene component [9,10].

CT scans with 3D software offer a more precise understanding of the patient-specific anatomy of the glenohumeral joint. The software can assist with preoperative planning to correct the abnormal version to a desired range of retroversion. While this software may improve the accuracy of glenoid component implantation and version correction, it is unknown if clinical outcomes are significantly improved with CT planning [11]. It also remains unknown how well preoperative planning correlates with intraoperative correction. These are two uncertainties that must be addressed as surgeons attempt to optimize intraoperative correction of glenoid version for the preservation of implant longevity.

It may be possible to use magnetic resonance imaging (MRI) for initial preoperative evaluation of glenoid version and assessment of any existing rotator cuff pathology [12]. However, for a true appreciation of the bone dysplasia of the glenoid, Farron et al. suggest that retroversion is optimally measured with CT before aTSA [13]. Thus, CT imaging is currently the standard imaging modality for bone evaluation and preoperative templating to assist intraoperative guidance. This Institutional Review Board (IRB)-approved prospective cohort study evaluated the correlation between glenoid version correction within the standard accepted range of 0 to 10 degrees and postoperative function in patients undergoing aTSA. The association of short-term clinical outcomes with glenoid version correction and the ability of surgeons to successfully implement the preoperative plan have not been previously reported [14]. Our hypothesis is that aTSA patients will have improved outcomes and patient satisfaction scores, and 3D CT planning will provide a reproducible method to restore the desired glenoid version.

This article was previously presented as a poster exhibit at the 2021 Orthopaedic Research Society (ORS) Annual Meeting from February 12 to 16, 2021.

## Materials and methods

This IRB-approved prospective cohort case series used consecutive sampling of patients undergoing primary aTSA at Northwestern Medicine Central DuPage Hospital, Department of Orthopaedics (Winfield, IL, USA) between 2018 and 2020. The sample size was determined by enrolling all consecutive eligible patients during the predefined study period. A formal statistical power calculation was not performed because this study was designed as a prospective cohort evaluating the reliability of CT-based glenoid version correction and associated short-term outcomes. Although the study was adequately powered to detect significant within-patient improvements over time, it was not specifically designed to detect small between-group differences based on glenoid version correction thresholds.

Fifty consecutive patients scheduled for aTSA with the principal investigator were screened and enrolled after completion of the informed consent process. Inclusion criteria were male and female patients with end-stage glenohumeral osteoarthritis who elected to undergo aTSA after failing traditional conservative measures of rest, activity modification, anti-inflammatory medications, and injection therapy, with clinical and radiographic evidence of a functional rotator cuff. Exclusion criteria included inflammatory arthropathies (e.g., rheumatoid arthritis), septic arthritis, inability to complete the consent process, full-thickness or high-grade partial rotator cuff tears (considered for reverse total shoulder arthroplasty [RTSA]), excessive glenoid retroversion (greater than 30 degrees), prominent B2 or B3 deformities, and substantial humeral head posterior subluxation (more than 80%).

At the preoperative and postoperative clinical appointments, patients’ enrollment eligibility was assessed by the study staff, and patients who met all inclusion and exclusion criteria were asked to participate in this study. Preoperative CT scans were performed in all enrolled patients, and the same 3D CT planning software (Blueprint^TM^, Tornier, Inc., Montbonnot-Saint-Martin, France) was consistently utilized to plan glenoid correction to restore anatomic glenoid version when needed. All CT scans were performed at the same institution, and all procedures were performed by the principal investigator. All procedures utilized stemless, cementless humeral fixation and cemented, pegged glenoid fixation and used the same implant manufacturer (Tornier).

Postoperative shoulder function was assessed in the clinic at two weeks, six weeks, 14 weeks, one year, and two years. At 14 weeks after surgery, patients had a repeat CT scan to evaluate component alignment and glenoid version. Two board-certified and fellowship-trained musculoskeletal radiologists interpreted all preoperative and postoperative CT scans with the conventional and vault methods, which are both accepted methods for measuring glenoid version values [15,16]. The radiologists were blinded as to the parameters and the objectives of the study.

**Figure 1:**
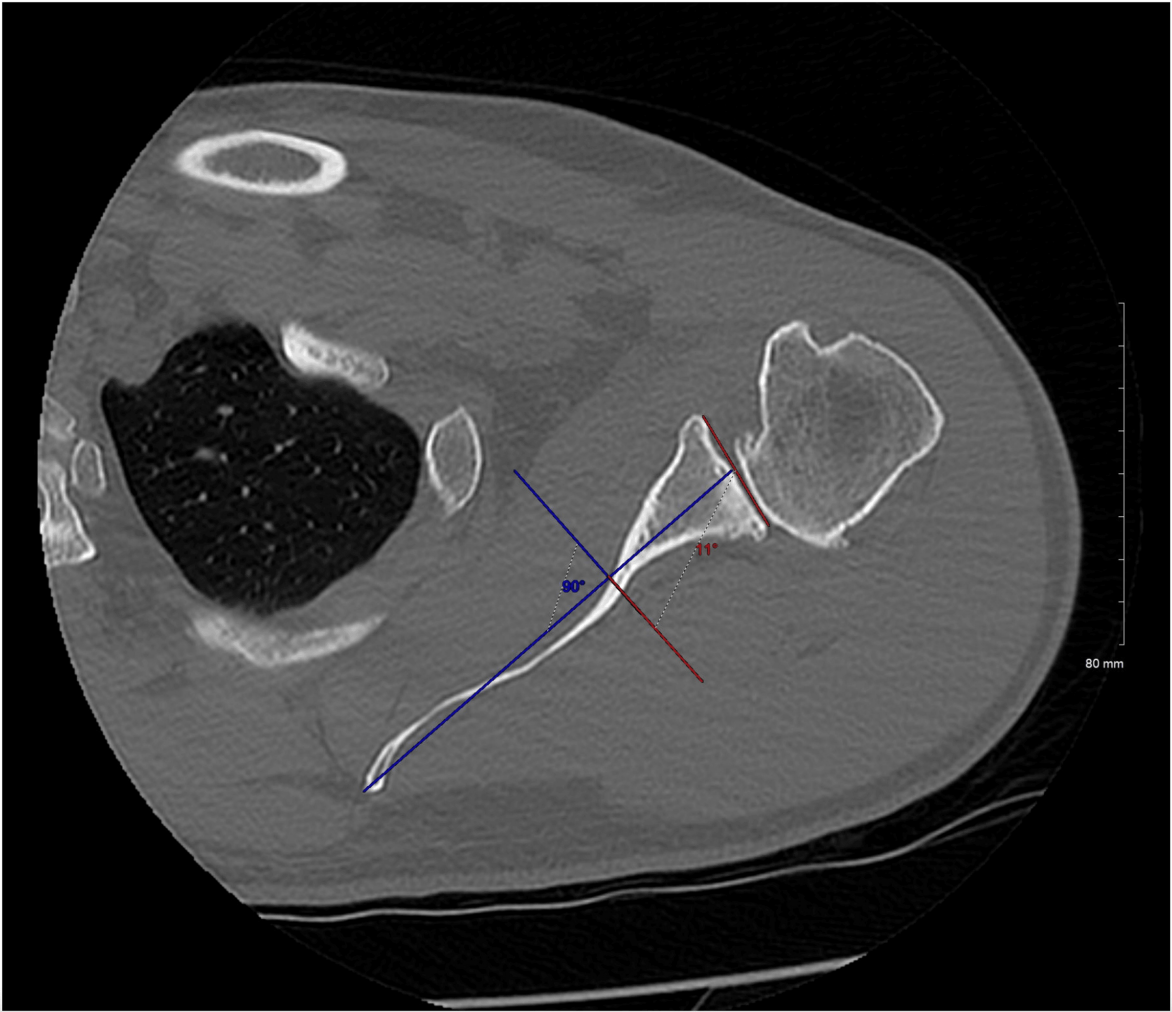
Conventional method for glenoid version calculation The conventional method for glenoid version calculates glenoid version as the angle between the line perpendicular to the scapular axis, defined as the line through the root of the scapular spine and the center of the glenoid, and the glenoid line, defined as the line connecting the anterior and the posterior rim of the glenoid. In this example, the glenoid version angle is 11 degrees (11 degrees retroversion).

**Figure 2:**
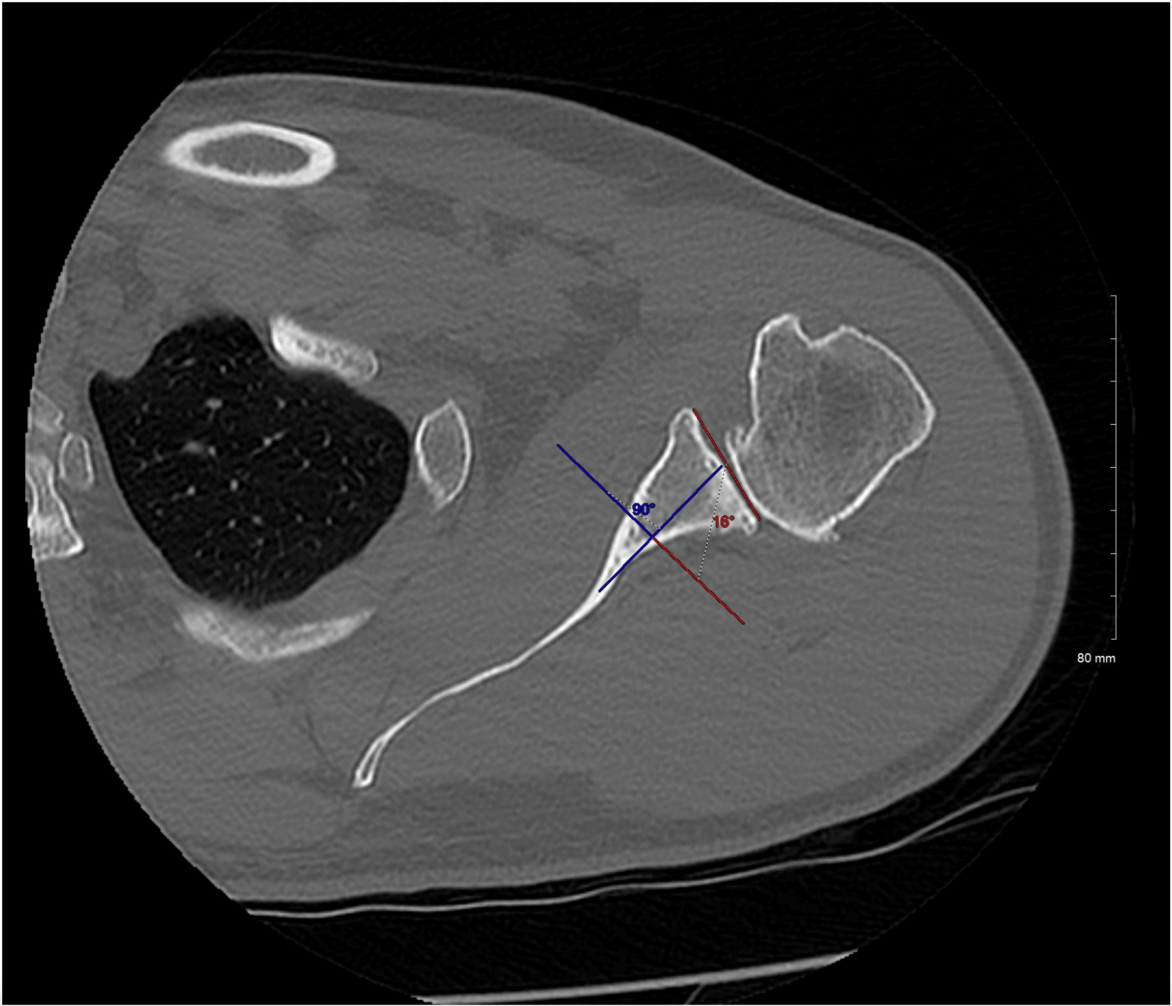
Vault method for glenoid version calculation The vault method for glenoid version calculates glenoid version as the angle between the line perpendicular to the vault axis, defined as the line connecting the tip of the scapular vault and the center of the glenoid line, and the glenoid line, defined by the line connecting the anterior and the posterior rim of the glenoid. In this example, the glenoid version angle is 16 degrees (16 degrees retroversion).

The glenoid line is defined in both reading methods as the line connecting the anterior rim with the posterior rim of the glenoid fossa. In the conventional method, the scapular axis is defined as the line connecting the tip of the medial border of the scapula and the center of the glenoid line. The glenoid version is then calculated as the angle between the glenoid line and the line perpendicular to the scapular axis (Figure 1). In the vault method, the glenoid vault axis is defined as the line connecting the tip of the scapular vault and the center of the glenoid line. The glenoid version is then calculated as the angle between the glenoid line and the line perpendicular to the glenoid vault axis (Figure 2).

The primary outcome in our study was patient-reported postoperative function as determined by the ASES (American Shoulder and Elbow Surgeons) shoulder score and PROMIS (Patient-Reported Outcomes Measurement Information System) Global Health survey, which were taken at baseline, 14 weeks, one year, and two years. The ASES shoulder score assesses pain levels, pain management, and the level of difficulty performing everyday tasks with each shoulder [17]. The PROMIS Global Health survey assesses mental health, physical health, and quality of life [18]. Both the ASES shoulder score and the PROMIS Global Health survey are free to use. In addition, range-of-motion measurements were taken at these intervals, including standing flexion, standing abduction, supine external rotation, supine internal rotation, Apley’s reach external rotation (hand behind head), and Apley’s reach internal rotation (hand behind back). In addition to subjective improvements in pain and functionality, shoulder range-of-motion measures are an objective measure of functional range of motion to assess for improvement and whether the range of motion is improved with more optimal glenoid version correction. The preoperative and postoperative measures were taken by the same member of the research team, who was also blinded to the type of prosthesis used as well as the glenoid version results of the preoperative and postoperative CT scans.

Percentages were used to compute the proportion of patients whose glenoid version correction was within the targeted normal range of less than 10 degrees of retroversion (0-9 degrees). We chose the target range of 0-9 degrees of retroversion for our preoperative templating as this range was felt to capture the normal range of retroversion. Those in and out of the normal range of glenoid version were compared to determine if true outcome measures varied between groups. To measure a statistically significant difference in preoperative and postoperative range of motion, a paired t-test was performed. An unpaired t-test was used to compare the postoperative range of motion between patients within and outside of the desired correction range. Statistical significance was set at p <0.05 for both t-tests.

## Results

Glenoid version

Of the 50 patients enrolled, 45 patients completed the study. Using the conventional and vault methods of measuring glenoid version values, 42% and 20% of patients had their preoperative glenoid version in the range of 0 to 10 degrees, respectively. Postoperatively, 76% and 62% of patients had their glenoid version corrected within the range of 0 to 10 degrees, respectively. With conventional values, seven patients (16%) had an anteversion correction below 0 degrees, and the remaining four patients (9%) had a retroversion correction above 10 degrees. With vault values, six patients (13%) had an anteversion correction below 0 degrees, and the remaining 11 patients (24%) had a retroversion correction above 10 degrees. Overall, 47% of the procedures utilized posterior polyethylene wedged implants. Of the 11 patients outside the normal correction range, four patients (36%) had posterior polyethylene wedges, and seven utilized asymmetric reaming techniques.

Range of motion

The differences between preoperative and postoperative standing flexion, standing abduction, supine external rotation, supine internal rotation, Apley’s reach external rotation, and Apley’s reach internal rotation means (Table 1) all demonstrated a statistically significant improvement in range of motion for each measurement (p < 0.0001). An unpaired t-test comparing these measurements within and outside the desired correction range of 0 to 10 degrees demonstrated no statistical significance (Table 2).

**Table 1:** Paired t-test for range of motion comparing the preoperative versus postoperative measurements ^a^Statistically significant.

Range-of-Motion Measurement	Preoperative (Mean)	Postoperative (Mean)	Difference Between Means	t-Value	p-Value	Estimated Standard Error
Standing flexion	110.38	151.29	40.91	11.03	<0.0001^a^	3.71
Standing abduction	96.11	137.67	41.56	8.06	<0.0001^a^	5.15
Supine external rotation	33.47	63.18	29.71	9.83	<0.0001^a^	3.02
Supine internal rotation	36.64	54.96	18.31	5.99	<0.0001^a^	3.06
Apley's reach external rotation	6.48	8.84	2.36	5.23	<0.0001^a^	0.45
Apley's reach internal rotation	9.64	13.50	3.86	8.15	<0.0001^a^	0.47

**Table 2:** Unpaired t-test for range-of-motion measurements comparing patients within versus outside the glenoid version correction range of 0-10 degrees

Range-of-Motion Measurement	Conventional Version Values			Vault Version Values		
	Within Correction Range (Mean)	Outside Correction Range (Mean)	p-Value	Within Correction Range (Mean)	Outside Correction Range (Mean)	p-Value
Standing flexion	149.06	158.18	0.14	148.68	155.59	0.21
Standing abduction	136.03	142.73	0.46	134.61	142.71	0.31
Supine external rotation	62.21	66.18	0.37	62.25	64.71	0.53
Supine internal rotation	54.50	56.36	0.67	53.32	57.65	0.26
Apley's reach external rotation	8.58	9.64	0.13	8.61	9.25	0.31
Apley's reach internal rotation	13.32	14.00	0.51	13.14	14.06	0.31

ASES shoulder score

There was a statistically significant decrease in the amount of pain medication taken, shoulder instability, and shoulder pain for patients two years postoperatively compared to their preoperative status (Table 3). An unpaired test comparing these parameters in patients within versus outside the glenoid version correction range did not show statistical significance in any measure. In addition, there was a statistically significant improvement in the two-year postoperative difficulty level of everyday task questions asked in the ASES shoulder score, such as lifting 10 pounds above the affected shoulder and sleeping on the affected side (Table 4).

**Table 3:** Paired t-test for ASES shoulder score questions regarding amount of pain medication taken, shoulder instability, and shoulder pain ASES, American Shoulder and Elbow Surgeons. ^a^Statistically significant.

ASES Shoulder Score Questions	Preoperative Response (Mean)	Postoperative Response (Mean)	Difference Between Means	t-Value	p-Value	Estimated Standard Error
Do you take pain medication (aspirin, Advil, Tylenol, etc)?	0.78	0.22	0.56	6.83	<0.0001^a^	0.08
Do you take narcotic pain medication (codeine or stronger)?	0.16	0.00	0.16	2.85	0.01^a^	0.06
Does your shoulder feel unstable (as if it is going to dislocate)?	0.38	0.00	0.38	5.17	<0.0001^a^	0.07
How unstable is your shoulder?	4.45	0.03	4.42	8.01	<0.0001^a^	0.55
How many pills do you take each day?	3.27	0.47	2.80	5.58	<0.0001^a^	0.50
How bad is your pain today?	4.98	0.69	4.29	10.96	<0.0001^a^	0.39

**Table 4:** Paired t-test to compare the preoperative versus postoperative responses of ASES shoulder score questions regarding the difficulty level of everyday tasks ASES, American Shoulder and Elbow Surgeons. ^a^Statistically significant.

ASES Shoulder Score Questions	Preoperative Response	Postoperative Response	Difference Between Means	t-Value	p-Value	Estimated Standard Error
Put on a coat	2.38	3.82	1.44	10.85	<0.0001^a^	0.13
Sleep on your painful or affected side	2.18	3.73	1.56	9.68	<0.0001^a^	0.16
Wash back/do up bra in back	1.41	2.98	1.57	8.22	<0.0001^a^	0.19
Manage toileting	3.11	3.84	0.73	5.11	<0.0001^a^	0.14
Comb hair	2.78	3.87	1.09	7.51	<0.0001^a^	0.15
Reach high shelf	1.93	3.76	1.82	12.14	<0.0001^a^	0.15
Lift 10 lb above the shoulder	1.86	3.66	1.80	9.67	<0.0001^a^	0.19
Throw a ball overhead	1.58	3.47	1.89	12.33	<0.0001^a^	0.15
Do usual work	2.62	3.80	1.18	10.17	<0.0001^a^	0.12
Do usual sport	1.91	3.68	1.77	11.66	<0.0001^a^	0.15

PROMIS Global Health survey

Two years postoperatively, there was a statistically significant increase in patients’ quality of life and ability to carry out everyday physical activities (p < 0.0001). Although not statistically significant, patients’ response regarding their physical health (Table 5) was trending toward improvement (p = 0.12).

**Table 5:** Paired t-test to compare the preoperative versus postoperative responses of PROMIS Global Health Survey questions PROMIS, Patient-Reported Outcomes Measurement Information System. ^a^Statistically significant.

PROMIS Global Health Survey Questions	Preoperative Response	Postoperative Response	Difference Between Means	t-Value	p-Value	Estimated Standard Error
In general, would you say your quality of life is?	3.96	4.31	0.36	2.43	0.02^a^	0.15
In general, how would you rate your physical health?	3.78	3.96	0.18	1.60	0.12^a^	0.11
To what extent are you able to carry out your everyday physical activities such as walking, climbing stairs, carrying groceries, or moving a chair?	4.09	4.67	0.58	3.67	0.0006^a^	0.16

Body mass index

The body mass index (BMI) average for all 45 patients was 30.6. The average BMI for patients outside the range of desired posterior version correction was 30.0. There was no significance of the ability to correct glenoid version within the range of normal to the patient’s BMI.

## Discussion

Preoperative CT scans provide valuable information to assist intraoperative decision-making regarding shoulder arthroplasty technique (anatomic versus reverse) and to guide glenoid version correction. The surgeon's success in implementing the preoperative plan to achieve the desired correction has yet to be published. This study is the first to report the accuracy of glenoid version correction and the associated short-term clinical outcomes following aTSA. Regardless of measuring glenoid version angles via the conventional or vault methods, more than 60% of the patients fell within the target range of 0 to 0 degrees. For those patients with glenoid correction outside of the desired range, a majority expressed improvement in their health and pain levels. With conventional values, 91% indicate their overall health, quality of life, and physical health as very good, if not excellent, and that percentage also expresses their 0-10 pain level as a 1 or below two years after surgery. Similarly, with vault values, 94% indicate their overall health, quality of life, and physical health as very good, if not excellent, and that percentage expresses their pain level on a scale of 0 to 10 to be 1 or below two years after surgery. Given a p-value <0.0001 for both standing flexion and supine external rotation angles, it can be concluded that aTSA has a statistically significant improvement in patients’ range of motion. As a result, it can be claimed, as well as verified with the results of the ASES shoulder scores, that patients can perform everyday tasks with minimal difficulty. Between patients within and outside the 0 to 10 degree correction range, the difference was not statistically significant. Thus, for a short-term two-year follow-up, patients perform similarly well in their range-of-motion measurements.

Over the past decade, the prevalence of shoulder arthroplasty cases has continued to grow at a substantial pace. Best et al. showed a nearly 50% aTSA case growth accompanied by a declining rate of hemiarthroplasties from 2012 to 2017 in the United States [19]. Anatomic shoulder arthroplasty remains the gold standard for end-stage osteoarthritis with an intact rotator cuff without severe glenoid deformity or humeral head subluxation. Loosening of the glenoid component is the most common culprit of aTSA failure [20]. In a review of nearly 3000 total shoulder arthroplasties, Bohsali et al. reported the incidence of aseptic loosening to be 39% after five years, with 83% of cases attributed to the failure of the glenoid component [9]. Shoulder arthroplasty in the setting of posterior glenoid bone loss and uncorrected version is associated with a threefold increase in stress in the cement mantle and a sevenfold increase in typical glenoid component micromotion [13]. Glenoid component retroversion can lead to eccentric loading with subsequent glenoid component loosening and failure [21]. While the importance of intraoperative version correction for implant survivorship is recognized, guidelines to treat excessive posterior glenoid wear have not been clearly established [22]. CT-based preoperative software offers surgeons a patient-specific plan, calculating values such as glenoid version and inclination. It also offers suggestions on implant sizing. This study evaluated the degree of reproducibility of the preoperative plan by studying implant alignment on postoperative CT scans.

The primary culprit for revision surgery in aTSA is aseptic loosening of the glenoid component [3,23]. Accurate positioning of the glenoid component and humeral components is essential for the longevity of the components. Using a retrospective medicare database of more than 9000 aTSA patients over 10 years, Cancienne et al. demonstrated that those with preoperative CT scans had a lower revision rate and that utilization of preoperative CT imaging grew significantly through the decade [24]. Raiss et al. found an 85% correlation of preoperative implant planning to implant selection at the time of surgery [23]. This illustrates that CT planning often accurately predicts implant sizing at the time of arthroplasty. Whether CT planning consistently improves surgeon accuracy in glenoid reconstruction has yet to be proven.

Recognition and correction of excessive posterior glenoid version to preserve component longevity is a critical step in the procedure. Historically, surgical options for excessive posterior glenoid version were limited to asymmetric anterior reaming or posterior bone grafting. New-generation posterior polyethylene wedge implants offer additional modalities for version correction. Eccentric reaming of the anterior glenoid is limited by the amount of bone that can be removed safely without risking glenoid vault perforation. Some authors recommend a preoperative retroversion limit of 15 degrees to correct with asymmetric reaming due to the concerns of bone loss and joint medialization [25]. Posterior corticocancellous bone graft is another option for treating larger posterior glenoid defects. However, this procedure is not only technically demanding but also presents a range of potential complications, including graft subsidence, resorption, and loosening in up to 30% of cases [22].

Posterior-augmented polyethylene glenoid components (posterior wedges) can adjust for posterior glenoid deficiency and minimize bone removal. Often chosen during the preoperative planning process, a wedge can restore the native joint line and prevent joint line medialization. Sabesan et al. showed that glenoid augments can more accurately correct glenoid version and prevent joint line medialization [26]. Ho et al. showed excellent clinical results and version correction in 71 patients with posterior augmented wedges for B2 and B3 glenoids [27]. Ianotti et al. showed similar clinical results and radiographic corrections comparing augmented stepped glenoids to standard non-wedge components [28]. While these studies show beneficial short-term results for wedge implants, no previous papers have confirmed the ability to correct version using posterior wedges and whether a successful correction equates to superior clinical outcomes.

This case-controlled IRB-approved cohort series shows short-term excellent outcomes with preoperative CT planning techniques for aTSA. All essential criteria for postoperative functional range of motion showed statistically improved measures postoperatively. Patients generally were very satisfied with their functionality and quality-of-life measures. Short-term measures do not show a statistically significant difference in shoulder range of motion or quality-of-life measures within and outside the range of normal glenoid version. Therefore, short-term outcomes do not appear to be impacted significantly by the restoration of normal glenoid version. The long-term implications of normal and abnormal postoperative version using polyethylene components require further long-term follow-up.

**Figure 3:**
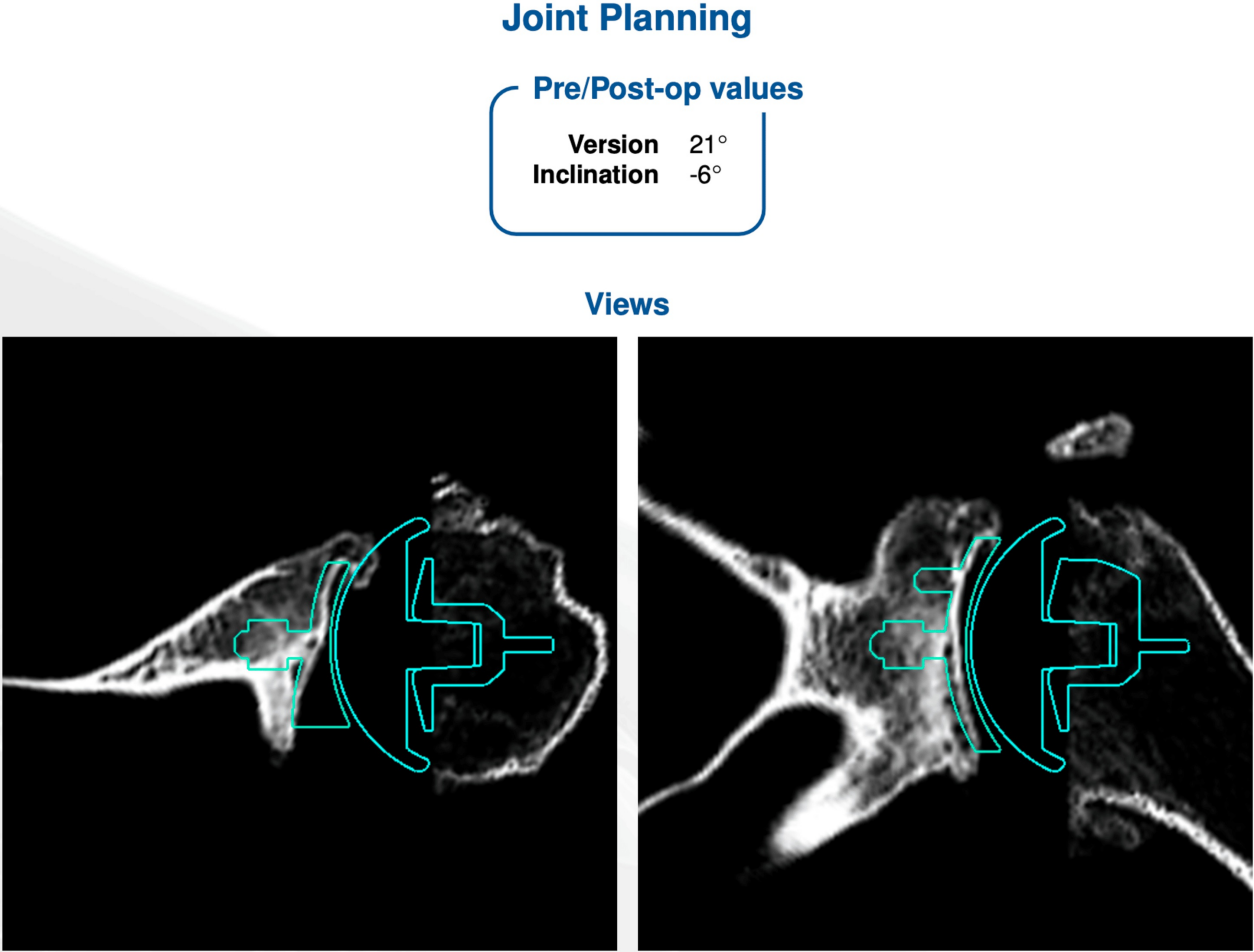
Example of CT scan planning prior to aTSA based on the patient’s preoperative version. A posterior wedge polyethylene implant had been chosen. aTSA, anatomic total shoulder arthroplasty.

While CT planning software assists in creating a template prior to the procedure, the correlation with postoperative anatomic glenoid version correction lacks excellent results in this study. Sixty percent of patients were corrected to within the normal range, and 40% were outside the range (0 to 10 degrees posterior version). All cases were templated with a preoperative CT scan to achieve a normal version with either asymmetric reaming or with a polyethylene posterior wedge (Figure 3). This suggests that while the technology helps with the plan, it does not consistently result in the anticipated postoperative version correction. Patient-specific factors such as BMI did not seem to affect correction success rates.

Advanced imaging modalities such as 3D CT help appreciate glenoid retroversion and posterior subluxation of the humeral head when considering an aTSA [29]. RTSA is an alternative for patients with rotator cuff insufficiency, severe glenoid deformities, and substantial posterior humeral head subluxation. Surgeons may intraoperatively opt for an RTSA due to an uncorrectable version or glenoid deformity in the setting of a Walch B2 or B3 glenoid [30]. While RTSA also continues to grow in utilization, aTSA remains preferred for surgical management of end-stage degenerative osteoarthritis where the humeral head is reasonably well centered in the glenoid fossa, the rotator cuff is intact, and the glenoid version is correctable to near-normal limits [19].

Limitations of this IRB-approved study include a lack of a control group (no preoperative planning and no CT scan), potential reporting bias from the radiologic interpretation, and potential patient reporting bias. While version correction was achieved with either corrective reaming or posterior augmentation, this heterogeneous population may alter functional outcomes due to medialization or lateralization of the glenoid. The study size did not allow for statistical analysis of the two groups.

## Conclusions

The focus of anatomic shoulder arthroplasty outcomes remains appropriate patient selection and restoration of glenoid version. The recent expansion and growth of the RTSA platform call into question whether diminishing criteria are evolving for aTSA. RTSA does have a well-documented complication rate generally believed to be higher than aTSA. aTSA generally produces a better postoperative range of motion (especially internal rotation) and fewer complications such as instability. Therefore, for patients who are candidates for an anatomic prosthesis, planning software will continue to offer surgeons the ability to use 3D CT imaging for preoperative planning. Implementing the preoperative plan to achieve a normal postoperative version will become a standard to measure the success of the plan. Technology offering real-time intraoperative feedback and virtual surgery is evolving. Future studies are needed to determine if real-time navigation will improve the accuracy of the preoperative plan. Validating planning technology through improved surgical outcomes will help advance the accuracy of shoulder arthroplasty.
